# Preparation of Vanadium Tailings-Based Ceramsite and Evaluation of Its Adsorption Performance for High-Fluoride Wastewater

**DOI:** 10.3390/ma19112201

**Published:** 2026-05-23

**Authors:** Jiangke Fan, Jing Huang, Yimin Zhang, Qian Wan, Nannan Xue

**Affiliations:** 1 School of Resource and Environmental Engineering, Wuhan University of Science and Technology, Wuhan 430081, China; fanjiangke___123@163.com (J.F.); zym126135@126.com (Y.Z.); wanqian@wust.edu.cn (Q.W.); cbdis@aliyun.com (N.X.); 2 State Environmental Protection Key Laboratory of Mineral Metallurgical Resources Utilization and Pollution Control, Wuhan University of Science and Technology, Wuhan 430081, China; 3 Collaborative Innovation Center of Strategic Vanadium Resources Utilization, Wuhan University of Science and Technology, Wuhan 430081, China; 4 Hubei Provincial Engineering Technology Research Center of High Efficient Cleaning Utilization for Shale Vanadium Resource, Wuhan University of Science and Technology, Wuhan 430081, China

**Keywords:** vanadium tailings, fly ash, porous ceramsite, fluoride removal, adsorption mechanism, solid waste utilization

## Abstract

**Highlights:**

- Ceramsite was produced using vanadium tailings as raw material.
- Ceramsite demonstrates a high level of efficiency in fluoride ion adsorption.
- Vanadium tailings-based ceramsite complies with national standards.

**Abstract:**

Vanadium tailings-based ceramsite (VT-Ceramsite), a type of porous ceramsite synthesized from vanadium tailings, was employed for the adsorption of fluoride ions from high-fluoride wastewater. This approach not only mitigates environmental pollution caused by industrial solid waste but also effectively removes fluoride contaminants from wastewater. The effects of vanadium tailings content, sintering temperature, and sintering time on the adsorption performance of the VT-Ceramsite were systematically investigated. Comprehensive characterizations via XRD, SEM, BET, and adsorption modeling reveal that fluoride sequestration by VT-Ceramsite is governed by the synergy between physical diffusion and chemical interactions. While the porous architecture provides essential transport pathways, the chemically active sites facilitate stable bonding. Future research will prioritize surface functionalization and tailoring strategies to augment the density of these active sites, thereby maximizing the adsorption potential for treating complex industrial effluents. The optimal preparation conditions were determined to be a ratio of 6.5:2.5:1 for vanadium tailings, fly ash, and kaolin, with a preheating temperature of 300 °C for 20 min and a sintering temperature of 900 °C for 20 min. In these conditions, the adsorption capacity for fluorine ions can reach 43.59 mg/g. VT-Ceramsite exhibited a specific surface area of 3.61 m^2^/g, hydrochloric acid solubility of 1.2%, and a void fraction of 48.68%, all parameters met national industrial standards. In addition, the leaching concentrations of heavy metals were found to be well below the limits specified in CJ/T 299-2008, indicating that the material poses no risk of secondary pollution. The study provides an economical, safe, and environmentally friendly route for the utilization of solid waste, and it offers a promising adsorbent for treating high-fluoride wastewater.

## 1. Introduction

Fluorine is a highly reactive non-metallic element whose distinctive chemical properties render it indispensable in a wide range of industrial applications, including hydrometallurgy, steelmaking, and chemical production [1,2]. China is endowed with abundant vanadium resources [3]. During the extraction process from vanadium shale, fluorine is commonly introduced as a leaching promoter. However, this practice generates large quantities of high-fluoride wastewater, which has the potential to lead to severe fluoride pollution [4]. Elevated concentrations of fluoride have been shown to pose significant risks to soil and water resources, ultimately threatening both ecological systems and human health [5,6]. Excessive fluoride intake has been linked to a range of diseases associated with fluorosis, including dental and skeletal fluorosis [7,8]. Consequently, the development of environmentally sustainable approaches for treating and mitigating fluoride pollution is of paramount importance.

The commonly employed fluoride removal techniques presently include adsorption, ion exchange, membrane separation, and chemical precipitation [9,10]. Among these treatment technologies, adsorption has been extensively employed due to its operational simplicity, cost-effectiveness, and high removal efficiency. The following adsorbents are commonly employed: activated alumina, bone char, clay minerals, and metal oxides [11,12]. However, conventional adsorbents are associated with several disadvantages, including limited adsorption capacity, high cost, and the potential risk of secondary pollution [13]. Hence, the development of novel fluoride adsorbents that are low-cost, highly efficient, and environmentally sustainable has become an important focus of current research. In this context, this study adopts a sustainable ‘waste-treat-waste’ strategy by transforming industrial wastes into ceramsite for fluoride remediation.

Vanadium tailings (VT) are defined as large-volume solid by-products that are generated during the extraction of vanadium from vanadium shale [10]. Massive stockpiling of VT not only leads to the wastage of land resources but also results in the leaching and dispersion of valuable metal elements with rainfall, causing resource loss and, in severe cases, water pollution [14]. At present, the resource utilization of VT is predominantly confined to the construction materials sector, while its overall comprehensive utilization efficiency remains relatively low [15]. In fact, VT is primarily composed of SiO_2_ and Al_2_O_3_, which makes it a suitable raw material for the preparation of ceramsite. The synthesis of ceramsite from complex industrial waste is fundamentally governed by the synergy of high-temperature phase transitions. During the high-temperature sintering process, partial decomposition of the raw components occurs, which in turn promotes the formation of abundant internal pores within the material [16], thereby endowing the ceramsite with a well-developed porous structure and enhanced specific adsorption capacity.

Despite the potential of vanadium tailings (VT) in ceramsite fabrication, their relatively low aluminum oxide (Al_2_O_3_)content often fails to meet the stoichiometric requirements defined by the Riley ternary phase diagram for optimal sintering [17]. To address this chemical deficiency, the incorporation of an aluminum-rich supplementary material is essential. In this context, fly ash (FA) is a prevalent industrial by-produce merges as an ideal candidate [18]. It is lightweight and has a small particle size, which makes it prone to airborne dispersion [19]. Furthermore, the presence of FA in soil and groundwater during periods of rainfall underscores the necessity for environmentally friendly treatment methodologies [20]. Since Al_2_O_3_ is the predominant component of FA, it can effectively compensate for the aluminum deficiency during the ceramsite production process [21]. Hence, the use of VT and FA as primary raw materials for VT-Ceramsite fabrication is theoretically justified.

In this study, VT-Ceramsite was strategically developed by leveraging the endogenous metal oxides within vanadium tailings (VT) to serve as inherent active sites for fluoride capture. Unlike conventional adsorbents, this work focuses on optimizing the sintering-induced synergy between VT and fly ash to create a robust matrix capable of handling extreme fluoride loads. Additionally, the adsorption mechanism was further elucidated through XPS analysis, specific surface area measurements, adsorption kinetics, and adsorption isotherm studies. This study demonstrates that VT-Ceramsite prepared from VT not only exhibits high fluoride adsorption efficiency but also achieves the dual goals of solid waste resource utilization and “treatment of wastes with processes of wastes against one another”, thereby contributing to sustainable and green development.

To ensure a transparent and rigorous experimental framework, the scope of this investigation is specifically defined by the systematic optimization of two pivotal fabrication variables: the raw material ratio and the sintering environment. Furthermore, the remediation of vanadium-bearing wastewater is significantly complicated by the intricate ionic matrix inherent in industrial effluents. Beyond the target fluoride ions, these aqueous systems typically harbor elevated concentrations of competing anions, most notably sulfates and chlorides, which are introduced during the leaching and acidification stages of vanadium extraction. The presence of these co-existing species can profoundly perturb the adsorption equilibrium through mechanisms such as competitive site occupancy or electrostatic interference. Consequently, this study incorporates experiments within multi-component solution systems to systematically evaluate the material’s chemoselective performance, ensuring its practical viability and robustness in authentic industrial scenarios.

## 2. Materials and Methods

### 2.1. Materials

Vanadium tailings (VT) and fly ash (FA) were utilized as the primary raw materials for ceramsite synthesis, with kaolin (KL) incorporated as a binding agent. The VT was obtained from a vanadium mine in Shanxi Province, China, and the FA was supplied by the Shijiazhuang Shang’an Power Plant (Huaneng International Power Co., Ltd., Shanghai, China). The primary chemical reagents utilized in this study, including sodium fluoride and sodium hydroxide, were of analytical reagent (AR) grade and were purchased from Sinopharm Chemical Reagent Co., Ltd. (Shanghai, China). All chemicals were used as received without further purification, and deionized water was employed for the preparation of all aqueous solutions.

### 2.2. Preparation of VT-Ceramsite

**Figure 1:**
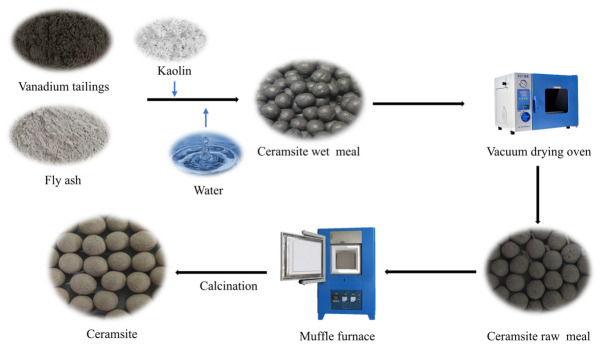
Flowchart of VT-Ceramsite preparation process.

To ensure both rapid and thorough reactions during sintering, the raw materials were initially subjected to vibratory milling for 5 min and then passed through a 200-mesh sieve [22]. The overall preparation process is illustrated in Figure 1. First, the raw materials were thoroughly mixed according to the specified proportions. Subsequently, 20% deionized water is added to the mixture to form raw ceramic granules with diameters of approximately 6–8 mm. The granules were dried at 105 °C for 2 h, followed by preheating at 400 °C for 20 min and subsequent sintering at 1000 °C for 20 min. Following a process of natural cooling, the final ceramsite product was obtained.

To determine the optimal preparation conditions for VT-Ceramsite, both the raw material ratios and sintering environment were systematically investigated. While the optimal mass ratio of raw materials (VT: FA: KL) was determined through single-factor experiments, the sintering environment was further optimized using L_9_(3^4^) orthogonal experimental design. The four critical factors investigated in this study included: preheating temperature, preheating time, calcination temperature, and calcination time.

### 2.3. Adsorption Experiments

Building upon the structural and compositional analysis of the VT-Ceramsite, its functional performance in fluoride removal was systematically evaluated through batch adsorption experiments. Batch adsorption experiments were conducted in 250 mL conical flasks at ambient temperature and pressure using a thermostatic shaker (120 rpm). Batch adsorption experiments were performed by placing a precise dosage of 10 g of VT-Ceramsite into 100 mL of synthetic fluoride solution with a known initial concentration of 4000 mg/L at an initial pH of 5.0 [4]. The solid-to-liquid ratio was maintained at 100 g/L throughout the optimization phase. To determine the adsorption isotherms, the initial fluoride concentrations were varied from 1000 to 6000 mg/L. Adsorption kinetics were investigated at different temperatures (298.15, 308.15, and 318.15 K) to understand the effect of temperature on the adsorption rate and to calculate kinetic parameters. To evaluate the selective adsorption performance of VT-Ceramsite in practical applications, competitive adsorption experiments were conducted using simulated high-fluoride wastewater. Considering the typical chemical composition of vanadium industrial effluents, the types and concentrations of co-existing ions (such as Ca^2+^, Mg^2+^, Cl^−^, ${\mathrm{SO}}_{4}^{2-}$) were selected and added to simulate real wastewater conditions. All batch experiments were maintained for 24 h to ensure the system reached full adsorption equilibrium. After adsorption, the solutions were filtered through 0.45 µm membranes, and the fluoride ion concentrations in the filtrates were determined using ion chromatography (IC). All adsorption experiments were conducted in triplicate to ensure data reliability, and results are reported as the mean values with standard deviations.

The fluoride removal efficiency (*R*) and adsorption capacity (*Q*) of the ceramsite were calculated using the following Equations (1) to (2):

(1) $$R=\frac{C_{0}-C_{e}}{c_{0}}\times 100\%$$

(2) $$Q=\frac{\left(C_{0}-C_{e}\right)V}{m}$$

where *C*_0_ (mg/L) and *C_e_* (mg/L) are the fluoride concentrations at the beginning and at equilibrium of the adsorption process; *V* (L) is the solution volume; *m* (g) is the dry mass of the ceramsite.

### 2.4. Reusability Experiments and Environmental Safety Assessment

To evaluate the regeneration potential and economic durability of the VT-Ceramsite, five consecutive adsorption–desorption cycles were performed. After the initial adsorption reached equilibrium, the fluoride-laden ceramsite was collected and treated with a 0.1 mol/L NaOH solution at a solid-to-liquid ratio of 1:50. The mixture was agitated for 120 min to facilitate the desorption of fluoride ions via hydroxyl ion exchange. Subsequently, the regenerated adsorbent was thoroughly rinsed with deionized water until the wash water reached a neutral pH and dried at 105 °C for subsequent cycles.

The raw materials utilized in ceramsite preparation, namely vanadium tailings and fly ash, contain alkaline substances and potentially harmful heavy metals. Excessive leaching of these metals poses environmental risks. To ensure the environmental compatibility of the VT-based ceramsite, leaching toxicity tests were conducted in accordance with the standard procedure GB 5085.3-2007 (Identification Standards for Hazardous Waste) [23]. Specifically, the extraction reagent consisted of a mixture of sulfuric acid and nitric acid (weight ratio of 2:1), with the pH adjusted to 3.20 ± 0.05 to simulate extreme acid rain conditions. The liquid-to-solid ratio was maintained at 10:1. The mixture was subjected to a rotary agitation apparatus at 30 rpm for 18 h at room temperature. Following the extraction, the concentrations of leached hazardous elements, specifically Cr, Cd, Pb, Cu, and Zn, were measured using ICP-OES (PerkinElmer, MA, USA).

### 2.5. Characterization

To systematically evaluate the physicochemical properties of the synthesized ceramsite, several characterization techniques were employed. X-ray diffraction (XRD, Rigaku D/MAX 2500PC, Takatsuki, Japan) was utilized to ascertain the crystalline phases of ceramsite. The microstructure and elemental composition of the ceramsite were examined using scanning electron microscopy (SEM, JEOL JSM-IT300, Tokyo, Japan) combined with energy-dispersive X-ray spectroscopy. Moreover, atomic force microscopy (AFM, SPM-9700HT, Shimadzu Corporation, Kyoto, Japan) was utilized to conduct a more comprehensive observation of the surface morphology and to analyze the surface roughness quantitatively. The specific surface area and pore size distribution of the ceramsite were measured using a Brunauer–Emmett–Teller analyzer (BET, ASAP2460, Beijing, China). To further investigate the adsorption mechanism and chemical environment, X-ray photoelectron spectroscopy (XPS, Thermo Fisher, East Grinstead, UK) was utilized to analyze the changes in the valence states and chemical bonding of key elements before and after fluoride adsorption. Regarding the aqueous phase analysis, the residual fluoride concentrations in the solutions following the adsorption experiments were precisely quantified using ion chromatography (IC, Metrohm 883, Herisau, Switzerland).

## 3. Results and Discussion

### 3.1. Characterization of Raw Materials

**Figure 2:**
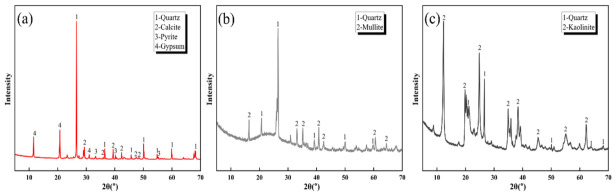
XRD patterns of VT (**a**), FA (**b**), and KL (**c**).

The chemical compositions of VT, FA, and KL were analyzed using X-ray fluorescence spectroscopy (XRF, Rigaku, Tokyo, Japan), as detailed in Table 1. As illustrated in Figure 2a, the XRD pattern of VT is dominated by the characteristic sharp peaks of quartz, which is consistent with the high SiO_2_ content (66.63%) identified in the XRF analysis. Minor diffraction peaks corresponding to calcite, pyrite, and gypsum are also observed, indicating a complex mineral composition. For Figure 2b, the presence of quartz and mullite is evident. The XRD pattern of Figure 2c reveals well-defined peaks of kaolinite and quartz, confirming its crystalline nature as a standard binding agent for ceramsite synthesis.

### 3.2. Effects of Preparation and Operating Parameters on the Fluoride Adsorption Capacity of VT-Ceramsite

#### 3.2.1. Raw Material Ratio

**Figure 3:**
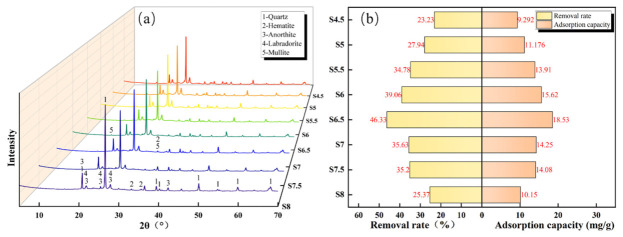
(**a**) XRD pattern of VT-Ceramsite; (**b**) removal rate and adsorption capacity of VT-Ceramsite.

As shown in Table 2, the raw material formulation design is presented in accordance with the Riley ternary phase diagram. Figure 3a shows the XRD pattern of the ceramsite after sintering. The primary crystalline phases identified in all samples include quartz, hematite, anorthite, labradorite, and mullite. The presence of hematite (Fe_2_O_3_) and aluminosilicate phases (mullite and anorthite) provides abundant active sites and surface hydroxyl groups, which are likely responsible for the superior fluoride removal efficiency observed in the S6.5 sample. Figure 3b shows the fluoride adsorption performance after 24 h of continuous exposure to a simulated fluoride-containing solution. The findings indicate that as the proportion of VT diminishes, the fluoride removal rate of the ceramsite initially rises, followed by a subsequent decline. Ceramsite exhibited the highest adsorption performance at a VT content of 65%, achieving a fluoride removal rate of 46.33% and an adsorption capacity of 18.53 mg/g.

#### 3.2.2. Sintering Environment

An orthogonal experiment was conducted based on the optimal raw material ratio to investigate the complex interactions within the thermal treatment process. An orthogonal experimental design was employed, encompassing four variables: preheating temperature, preheating time, sintering temperature, and sintering time. The experimental outputs were subjected to Analysis of Variance to quantify the statistical significance of each thermal parameter. The experimental design and results are presented in Table 3.

**Figure 4:**
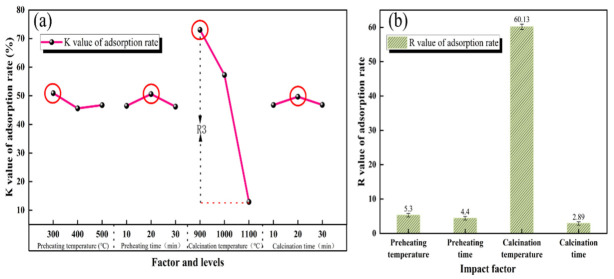
K (**a**) and R (**b**) values for different influencing factors.

As shown in Figure 4a, the VT-Ceramsite exhibited the highest removal rate at a sintering temperature of 900 °C, with an adsorption constant (K) of 73.06. The lowest K value (12.93) was observed at 1100 °C. K values represent the average adsorption capacity for each factor at a specific level, which determines the optimal parameter settings. The R value is defined as R = K_max_ − K_min_, where a larger R signifies a more dominant effect of that factor on the fluoride removal process. This suggests that, within a certain specific temperature range, VT-Ceramsite prepared at lower sintering temperatures exhibits superior adsorption performance; increasing the temperature results in a decline in adsorption capacity. This phenomenon is attributed to the partial melting of the glassy phase and the closure of internal pores at high temperatures, which significantly reduces the available surface adsorption sites and hinders physical adsorption [24].

Figure 4b shows that the influence of sintering conditions on adsorption performance is in the following order: calcination temperature > preheating temperature > preheating time > calcination time. Therefore, optimizing the sintering environment is essential for maximizing the adsorption capacity of ceramsite.

#### 3.2.3. Optimized Sintering Conditions for VT-Ceramsite Fabrication

**Figure 5:**
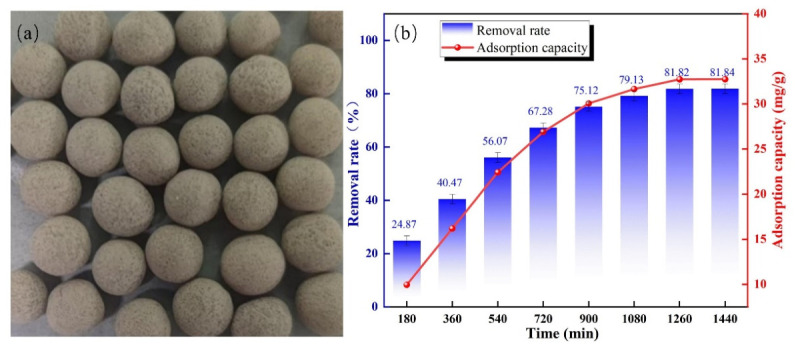
(**a**) Appearance of ceramsite prepared under optimal conditions; (**b**) The corresponding fluoride removal rate and adsorption capacity.

Following a comprehensive consideration of all experimental factors, the optimal preparation conditions for VT-Ceramsite were determined as follows: a mass ratio of VT, FA, and KL of 6.5:2.5:1, a preheating temperature of 300 °C for 20 min, and a sintering temperature of 900 °C for 20 min. Continuous adsorption experiments were conducted under conditions identical to those of the preceding experiments, as illustrated in Figure 5b. Following 24 h, the removal rate of fluoride by the ceramic granules was found to be 81.84%, with an adsorption capacity of 32.73 mg/g. This performance was significantly superior to that of any other ceramic granule sample within the experimental group.

Figure 5a shows the appearance of the ceramsite prepared under the above-mentioned parameters. The physical properties of the subject were evaluated in accordance with the relevant criteria of GB/T 17431.2-2010 [25] and CJ/T 299-2008 [26]. As shown in Table 4, all measured properties of the prepared VT-Ceramsite meet the relevant standards, indicating its satisfactory performance and compliance with the fundamental requirements for water treatment applications.

#### 3.2.4. Effect of Coexisting Ions on Adsorption Performance

**Figure 6:**
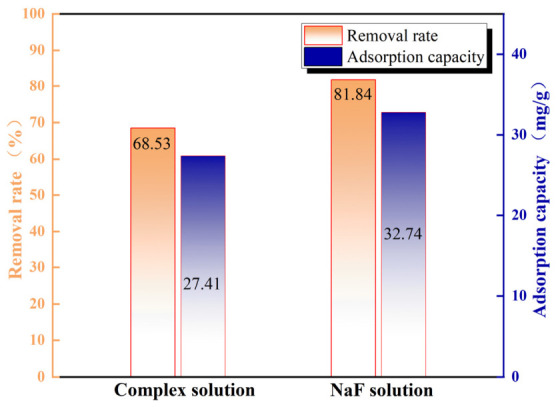
Comparison of the adsorption performance of VT-Ceramsite in different solution environments.

VT-Ceramsite prepared in this study exhibited good fluoride adsorption capacity in NaF solutions. However, the presence of coexisting ions such as Al^3+^, Ca^2+^, Mg^2+^, ${\mathrm{SO}}_{4}^{2-}$, and Cl^−^ significantly affects the adsorption performance of the ceramsite towards fluoride ions in actual high-fluoride wastewater generated from the vanadium processing industry. Adsorption tests using high-fluoride wastewater (Table 5) demonstrated that the equilibrium fluoride adsorption capacity after 24 h was 27.41 mg/g, which is lower than the saturation capacity observed in pure NaF solution (Figure 6). The significant attenuation in adsorption performance observed in the complex solution can be elucidated by a dual inhibitory mechanism involving aqueous speciation and surface site competition. Firstly, the presence of dissolved aluminum and silicon species facilitates the formation of stable Al-F and Si-F complexes in the aqueous phase [27], which decreases the concentration of free fluoride ions available for adsorption by the VT-Ceramsite, thereby lowering the adsorption capacity. Secondly, the coexisting anions within the multi-component matrix exert competitive pressure by occupying the limited active sites on the ceramsite surface. This competitive adsorption further hinders the accessibility of fluoride ions to the Ca/Al functional groups, ultimately resulting in the observed reduction in total fluoride uptake under realistic industrial conditions [28]. The coexistence of these ions within actual wastewater has been demonstrated to affect the fluoride adsorption efficiency of VT-Ceramsite; nevertheless, VT-Ceramsite has been shown to exhibit a certain degree of fluoride removal capability.

### 3.3. Characterization and Adsorption Mechanism of VT-Ceramsite

#### 3.3.1. Microstructural Characterization of the Surface

**Figure 7:**
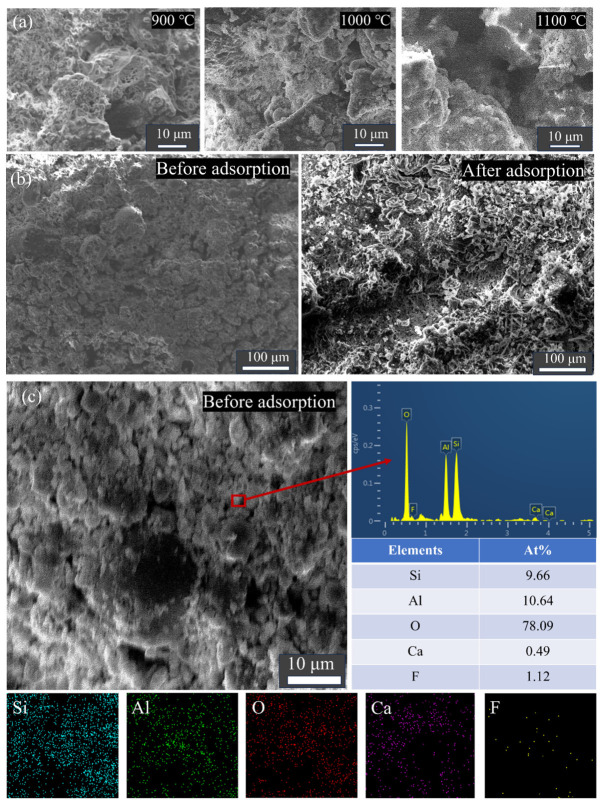

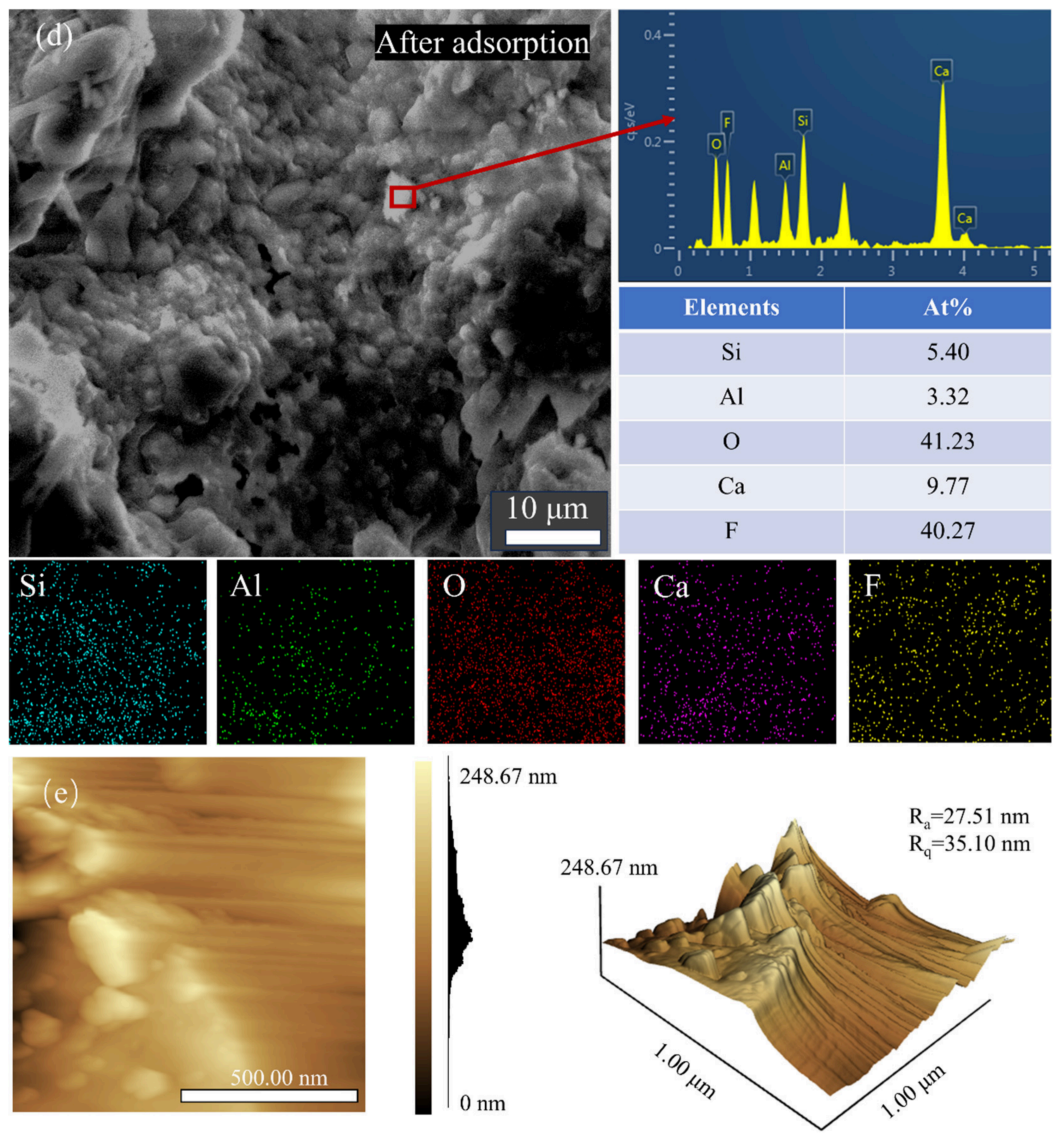
(**a**) SEM images of the VT-Ceramsite surface; (**b**–**d**) SEM-EDS images of the VT-Ceramsite before and after fluoride adsorption; (**e**) AFM images of the VT-Ceramsite.

Figure 7a presents the SEM images of the ceramsites sintered at different temperatures. At 900 °C, the surface consists of densely packed and interconnected granular clusters that exhibit a rough morphology with numerous microscale protrusions and depressions. These features collectively provide a high density of accessible adsorption sites and a large specific surface area. When the temperature increased to 1000 °C, the boundaries of the fine particles became smoother, and local melting traces appeared on the surface. At 1100 °C, the surface became markedly denser and smoother, with large closed pores or voids formed by the coalescence of molten pores, and the particle surface was coated with a continuous melted layer.

Figure 7b–d shows the SEM-EDS images of the VT-Ceramsite sintered at 900 °C before and after fluoride adsorption. Before adsorption, the VT-Ceramsite exhibits a relatively porous surface structure; however, after adsorption, the surface becomes significantly coarser with the appearance of dense flocculent aggregates, providing direct visual evidence of fluoride entrapment. The significantly increased fluoride signal confirmed its effective adsorption of fluoride ions [29]. Figure 7e shows that the surface roughness of the ceramsite is 27.51 nm, the pores were well-distributed and interconnected, with numerous fine channels and micro-cracks observed on the surface.

#### 3.3.2. XRD and BET Analysis

**Figure 8:**
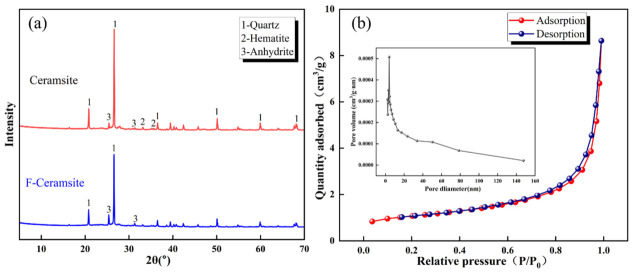
(**a**) XRD images of VT-Ceramsite before and after adsorption; (**b**) BET images of VT-Ceramsite.

As Figure 8a shows, the dominant crystalline phases in the sintered products are quartz, hematite, and anhydrite. Unlike the raw materials, the diffraction peaks of pyrite and kaolinite have disappeared completely, indicating their complete decomposition during sintering. After the adsorption process, no diffraction peaks that are indicative of crystalline fluoride compounds were detected. This finding suggests that fluorine fixation by the ceramic granules does not involve the formation of new crystalline phases. Instead, it is more probable that fluorine is immobilized on the adsorbent via surface complexation or precipitation mechanisms, thereby forming an amorphous coating on the granule surface that attenuates the diffraction signals [30].

Figure 8b shows the results of N_2_ adsorption–desorption isotherm tests conducted on the prepared ceramic aggregate samples. The N_2_ adsorption–desorption isotherms exhibit typical Type III characteristics, reflecting a well-developed mesoporous structure that serves as the physical foundation and transport pathways for adsorption [31]. With an average pore size of 14.74 nm and a pore size distribution ranging from 2 to 50 nm, the ceramsite is classified as a typical mesoporous material. Its most probable pore size is 3.94 nm, and its specific surface area is 3.61 m^2^/g.

#### 3.3.3. XPS Analysis

**Figure 9:**
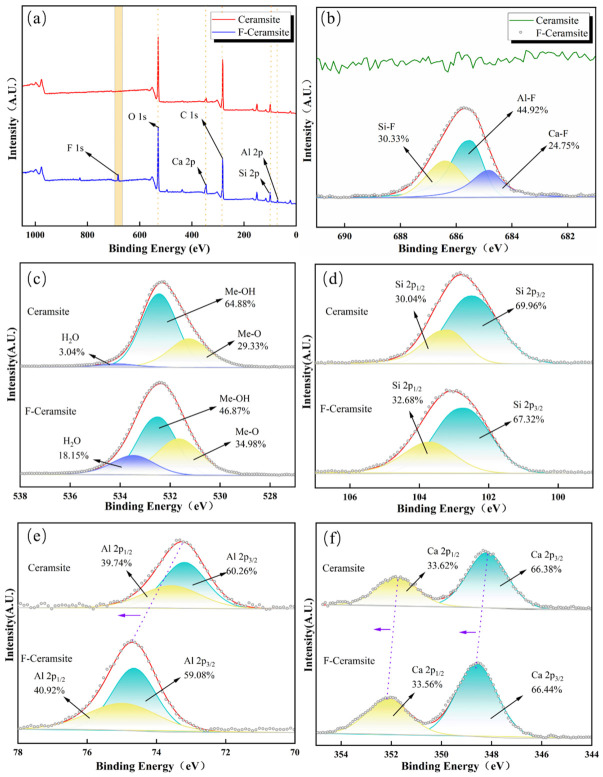
(**a**) XPS spectra of the before and after VT-Ceramsite adsorption treatment; (**b**) F 1s, (**c**) O 2p, (**d**) Si 2p, (**e**) Al 2p, (**f**) Ca 2p spectra of XPS before and after adsorption.

The XPS analysis results (Figure 9) further confirmed the fluoride adsorption behavior. As demonstrated in the complete survey spectra (Figure 9a), a discernible F 1s signal became apparent following the adsorption process, thereby signifying the effective immobilization of fluoride ions onto the VT-Ceramsite. In order to elucidate the chemical environment of fluoride and its interaction with surface-active metal sites, the F 1s, O 1s, Si 2p, Al 2p, and Ca 2p spectra were deconvoluted using Gaussian–Lorentzian fitting (Figure 9b–f). The results indicate that the F 1s spectrum can be deconvoluted into three distinct peaks: 684.8 eV, which is attributed to the Ca-F bond; 685.5 eV, which is associated with the Al-F bond; and 686.4 eV, which corresponds to the Si-F bond. The O 1s spectrum is composed of three components assigned to Metal-O, Metal-OH, and H_2_O species. Following the adsorption process, the proportion of metal-OH decreased from 64.88% to 46.87%, indicating that surface hydroxyl groups played an active role in the fluoride adsorption process. Concurrently, both Al 2p and Ca 2p exhibited an evident positive shift in binding energy, implying the formation of stable metal-fluorine (Metal-F) bonds through chemical coordination with surface-active sites [32]. Based on the chemical shifts observed in the F 1s spectrum, it is suggested that fluoride fixation on the ceramsite surface could occur through coordination with elements such as Al, Ca, and Si. This further corroborates that the exchange of fluoride ions with surface hydroxyl groups, followed by complexation with metal active centers, constitutes one of the key mechanisms governing fluoride removal by the ceramsite.

#### 3.3.4. Adsorption Isotherm Models

To further investigate the adsorption performance and mechanism of the VT-Ceramsite toward fluoride, batch experiments were conducted under a fixed adsorbent dosage of 100 g/L, a contact time of 1440 min, and a temperature of 298.15 K, while varying the initial fluoride ions from 1000 mg/L to 6000 mg/L. The relationship between the equilibrium adsorption capacity and the equilibrium concentration of fluoride in solution was analyzed, and the experimental data were described and fitted using the Langmuir [33] and the Freundlich [34] isotherm models.

Langmuir model:

(3) $$Q_{e}=\frac{Q_{m}K_{L}C_{e}}{1+K_{L}C_{e}}$$

Freundlich model:

(4) $$Q_{e}=K_{F}C_{e}^{\frac{1}{n}}$$

where *Q_e_* (mg/g) is the equilibrium adsorption capacity of the VT-Ceramsite, *Q_m_* (mg/g) is the maximum saturated adsorption capacity, *C_e_* (mg/L) denotes the equilibrium fluoride concentration in solution, *K_L_* is the Langmuir constant, and *K_F_* and *n* are the Freundlich constants.

**Figure 10:**
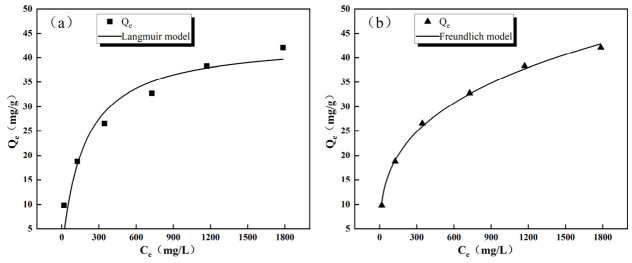
(**a**) Langmuir model; (**b**) Freundlich model.

As shown in Figure 10, the adsorption capacity of the VT-Ceramsite toward fluoride increases with increasing initial concentration. As summarized in Table 6, the experimental data are better fitted by the Freundlich model, with a correlation coefficient of 0.9971, which is higher than that of the Langmuir model. The Freundlich model applies to multilayer adsorption on heterogeneous surfaces, indicating that adsorption sites with different energy levels coexist on the adsorbent surface, leading to varied adsorption interactions with the adsorbate. In addition, the Freundlich adsorption intensity index (1/*n*) is 0.3067. A value of 1/n in the range of 0.1–0.5 is generally considered indicative of a favorable adsorption process.

#### 3.3.5. Adsorption Kinetics Models

To identify whether the rate-controlling step of fluoride adsorption is dominated by diffusion or surface binding reactions, kinetic experiments were conducted under the following conditions: an adsorbent dosage of 100 g/L, an initial fluoride concentration of the NaF solution of 4000 mg/L, a temperature range of 298.15–318.15 K, and a contact time of 1440 min. The obtained data were subsequently fitted and compared using the pseudo-first-order [35] and pseudo-second-order [36] kinetic models to evaluate their applicability and to elucidate the underlying rate-controlling mechanism. The mathematical expressions of the two models are given as follows.

Pseudo-first-order model:

(5) $$\ln\left(Q_{e}-Q_{t}\right)=\ln Q_{e}-k_{1}t$$

Pseudo-second-order model:

(6) $$\frac{t}{Q_{t}}=\frac{1}{k_{2}Q_{e}^{2}}+\frac{t}{Q_{e}}$$

where *k*_1_ (min^−1^) is the pseudo-first-order rate constant, *k*_2_ (min^−1^) is the pseudo-second-order rate constant, *Q_t_* (mg/g) denotes the adsorption capacity at time *t*, *Q_e_* (mg/g) is the equilibrium adsorption capacity, and *t* (min) represents the contact time.

The rate-controlling step of fluoride adsorption by the ceramsite can be investigated using the intraparticle diffusion model [37], which is expressed by the following equation:

(7) $$Q_{t}=k_{p}t^{\frac{1}{2}}+C$$

where *k_p_* (mg·g^−1^·min^1/^^2^) is the intraparticle diffusion rate constant, *Q_t_* (mg/g) is the adsorption capacity at *t*, and *t* (min) represents the contact time.

**Figure 11:**
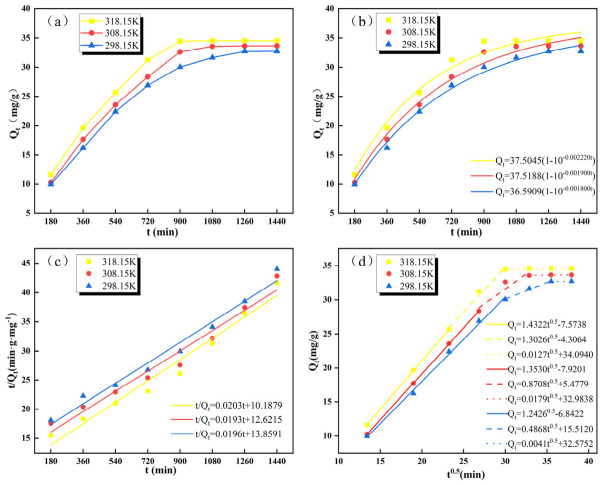
(**a**) Adsorption kinetics of fluorine by VT-Ceramsite; (**b**) Pseudo-first-order kinetic model; (**c**) Pseudo-second-order kinetic model; (**d**) Intra-particle diffusion model.

The adsorption kinetics of fluoride onto the ceramsite at 298.15–318.15 K are presented in Figure 11a. The adsorption capacity increased sharply during the initial 0–900 min, followed by a slower increase after 1080 min, and equilibrium was essentially reached at 1260 min.

The pseudo-first-order and pseudo-second-order kinetic models are illustrated in Figure 11b,c, with the corresponding parameters summarized in Table 7. The pseudo-first-order model exhibited higher correlation coefficients than the pseudo-second-order model across all temperatures, and the calculated equilibrium adsorption capacities were closer to the experimental values. This indicates that ceramsite’s porous framework serves as a vital conduit, facilitating the physical diffusion and transport of fluoride ions into the interior. And this physical aspect persists throughout the process. It should be noted that the K values obtained in this study reflect the intrinsic kinetic behavior of the unmodified VT-Ceramsite. While the adsorption rate is moderate compared to highly engineered nano-adsorbents, this research serves as a preliminary feasibility assessment of transforming raw solid waste into functional ceramics.

The intraparticle diffusion model was further applied to analyze the adsorption mechanism (Figure 11d, Table 8). The high R^2^ values for the first and second stages (0.7972–0.9999) indicate that the adsorption process is primarily controlled by both liquid-phase diffusion and intraparticle diffusion. However, as the fitted lines for all three stages do not pass through the origin, it is possible that other mechanisms also contribute to the overall adsorption process.

#### 3.3.6. Adsorption Mechanism

**Figure 12:**
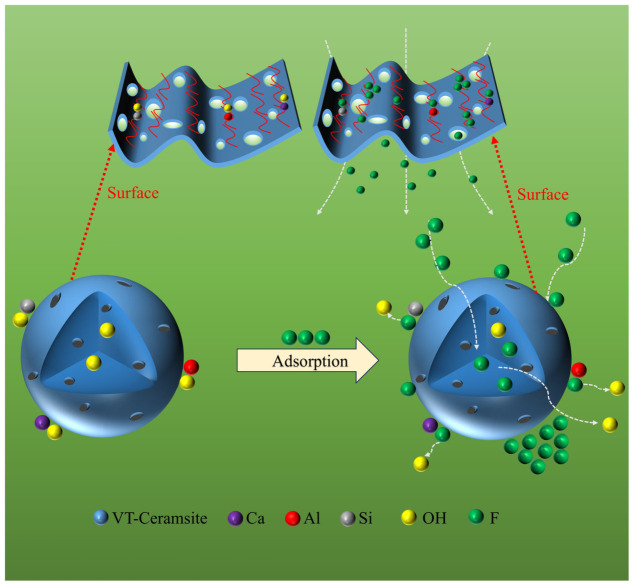
Adsorption mechanism of fluoride on VT-Ceramsite.

As illustrated in Figure 12, fluoride removal by VT-Ceramsite is driven by a synergetic physical and chemical adsorption process. The heterogeneous porous structure provides the essential transport pathways and physical foundation, enabling fluoride ions to penetrate into the internal channels. Concurrently, chemical adsorption occurs on the active sites to ensure the stable immobilization of fluoride. Based on the direct experimental evidence from XPS analyses, the removed fluoride is definitively sequestered on the solid phase, demonstrating that the overall process is governed by the combination of physical accessibility and surface-bound chemical interactions.

### 3.4. Reusability of VT-Ceramsite

**Figure 13:**
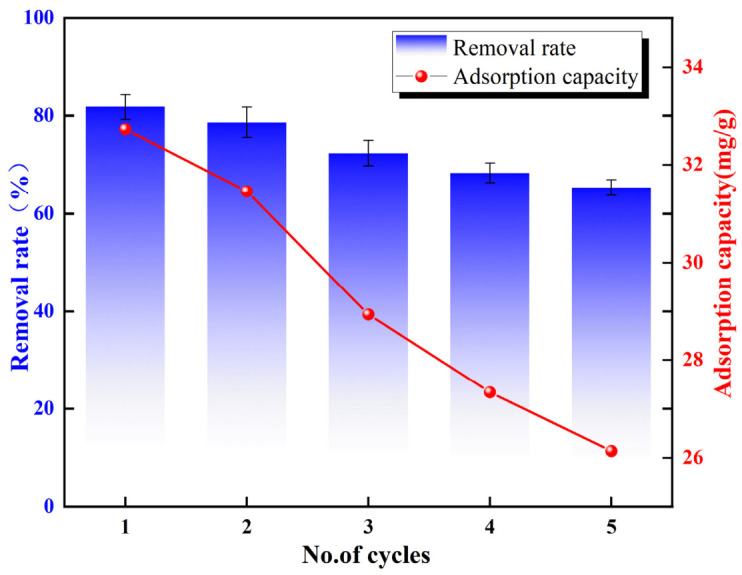
Effect of cyclic adsorption times on the adsorption of VT-Ceramsite.

To evaluate the sustainability of the adsorbent, the regeneration potential of the fluoride-rich VT-Ceramsite was systematically evaluated in Figure 13. Preliminary regeneration experiments, utilizing a 0.1 mol/L NaOH solution as the desorbing agent, ref. [38] demonstrated that the ceramsite retains 88.42% of its initial adsorption capacity after three consecutive adsorption–desorption cycles. Although a gradual decline in removal efficiency was observed as the number of cycles increased, the material still retained a substantial adsorption capacity of 26.14 mg/g even after five cycles. This reduction is primarily ascribed to the synergistic effect of active site deactivation and pore obstruction. On the one hand, the repetitive exposure to the NaOH regenerant facilitates the trace dissolution of surface-active Ca and Al components, thereby diminishing the chemical affinity for fluoride. On the other hand, the accumulation of residual fluoride or mineral precipitates within the internal pores causes irreversible blockage, limiting the accessibility of the remaining active sites. Despite this decline, the material retains a significant portion of its initial capacity, demonstrating a reasonable degree of reusability for industrial applications where cost-effective waste-based adsorbents are preferred.

### 3.5. Environmental Safety Analysis of VT-Ceramsite

The leaching concentrations of heavy metals in the ceramsite were evaluated in accordance with the GB 5085.3-2007 “Identification Standards for Hazardous Waste”, and the results are presented in Table 9. The leaching concentrations of all heavy metals are well below the national regulatory limits. This phenomenon is plausibly attributable to the high-temperature calcination process, during which heavy metals are incorporated into the glassy phase and form stable solid solutions, thereby achieving effective immobilization [21]. Consequently, the utilization of VT for ceramsite production not only facilitates the recovery of waste materials but also prevents the occurrence of secondary environmental pollution, thereby suggesting that the resulting ceramsite is a comparatively safe adsorbent.

### 3.6. Literature Contrast Analysis

Table 10 compares the fluoride adsorption performance of various adsorbents reported in the literature (mainly solid waste-derived materials) with that of the ceramsite prepared in this study. Notably, the VT-Ceramsite synthesized in this work represents a sustainable and high-value strategy for the resource utilization of vanadium extraction tailings, effectively transforming hazardous solid waste into a functional environmental material.

The Langmuir isotherm yielded a monolayer adsorption capacity (Q_L_) of 43.5932 mg/g. However, since the Langmuir model’s homogeneous monolayer adsorption assumption does not fully represent the actual system, the resulting value is primarily theoretical. In contrast, the Freundlich isotherm (Table 5) provided a better fitting performance than the Langmuir model. The maximum equilibrium adsorption capacity obtained experimentally was 42.1544 mg/g under an initial fluoride concentration of 6000 mg/L. Therefore, both the empirical Freundlich parameter (Q_F_) and the theoretical Langmuir parameter (Q_L_) are presented in Table 9 for comparison. The results demonstrate that the VT-Ceramsite synthesized from vanadium-extraction tailings exhibits a higher adsorption capacity than most unmodified solid waste-based adsorbents, and still shows a distinct advantage over several modified materials.

In summary, while this study demonstrates a promising maximum fluoride adsorption capacity, it also identifies certain kinetic limitations inherently associated with the unmodified, sintered nature of the tailings-based matrix. Nonetheless, this work establishes a critical scientific baseline for the valorization of vanadium tailings, proving their fundamental feasibility as a functional substrate. While the current study focuses on the inherent efficacy of the raw tailings, there is substantial potential to further optimize the adsorption kinetics and equilibrium capacity through targeted surface functionalization in future work, highlighting its strong potential as an environmentally friendly and high-performance adsorbent for fluoride-containing wastewater treatment.

## 4. Conclusions

This study demonstrates a sustainable and economically viable “waste-to-resource” strategy by transforming hazardous vanadium-extraction tailings (VT) into high-performance porous adsorbents. The following major insights summarize the scientific and practical significance of this work:

(1) The optimal adsorption performance of the VT-Ceramsite was achieved when the mass ratio of VT, FA, and KL was 6.5:2.5:1, with a preheating temperature of 300 °C for 20 min and a calcination temperature of 900 °C for 20 min. While the synthesis of VT-Ceramsite involves a high-temperature calcination process, the associated thermal costs should be balanced against the significant environmental and economic savings achieved through large-scale solid waste management. Based on the Langmuir model, the theoretical fluoride removal capacity of the prepared ceramic pellets is 43.59 mg/g, surpassing the capacities of most solid waste-based fluoride removal agents reported in the literature. This work validates the fundamental feasibility of using unmodified VT as a primary raw material for functional adsorbents, achieving the goal of high-volume solid waste consumption.
(2) VT-Ceramsite exhibited a specific surface area of 3.61 m^2^/g, solubility in hydrochloric acid of 1.2%, and a void fraction of 48.68%, meeting the national standard for artificial ceramsite filter materials used in water treatment. The leaching concentration of heavy metals in the ceramic granules falls below national standard limits, indicating no risk of secondary environmental pollution.
(3) During the fluoride adsorption process by ceramsite, distinct complexation effects involving Si-F, Al-F, and Ca-F are observed, which contribute to an enhanced adsorption performance. The adsorption behavior conforms well to the Freundlich isotherm and the pseudo-first-order kinetic model, which is attributed to the complex morphology of the VT-Ceramsite; the distinct differences between the internal pore structures and surface rugosity result in a wide distribution of adsorption sites with varying energy levels. In summary, the removal of fluoride by VT-Ceramsite is a complex, multi-mechanistic process synergistically driven by physical diffusion and chemical complexation between fluoride ions and surface metal-active sites.
(4) Preliminary investigations confirm that VT-Ceramsite possesses exceptional resistance to the interference of coexisting ions, maintaining a high fluoride adsorption capacity in diverse ionic environments. This inherent selectivity confirms the material’s practical utility for treating multi-component industrial wastewater. Furthermore, while the current batch experiments establish the high intrinsic capacity of VT-Ceramsite, subsequent investigations using continuous fixed-bed columns are essential to evaluate breakthrough curves and operational longevity, providing the necessary engineering data to scale up the treatment process for industrial applications.

## Figures and Tables

**Table 1:** Typical chemical composition of VT, FA, and KL (wt.%).

Material	SiO_2_	Al_2_O_3_	Fe_2_O_3_	CaO	MgO	Na_2_O	V_2_O_5_
VT	66.63	2.54	2.38	6.26	0.31	0.44	0.12
FA	43	23	2.5	5.6	0.95	0.66	-
KL	52.15	42.01	1.25	0.099	0.3	0.072	-

**Table 2:** Raw material formula of VT, FA, and KL.

Sample	VT (wt.%)	FA (wt.%)	KL (wt.%)
S8	80	10	10
S7.5	75	15	10
S7	70	20	10
S6.5	65	25	10
S6	60	30	10
S5.5	55	35	10
S5	50	40	10
S4.5	45	45	10

**Table 3:** Program design of the sintering environment.

NO	Preheating Temperature (°C)	Preheating Time (min)	Calcination Temperature (°C)	Calcination Time (min)	Adsorption Rate (%)
1	300	10	900	10	73.96
2	300	20	1000	20	65.20
3	300	30	1100	30	13.61
4	400	10	1000	30	52.94
5	400	20	1100	10	12.66
6	400	30	900	20	71.27
7	500	10	1100	20	12.53
8	500	20	900	30	73.96
9	500	30	1000	10	53.75

**Table 4:** Various parameters of the VT-Ceramsite products.

	Standard	Product
Grain diameter (mm)	0.5–9.0	6.0–8.0
Apparent density (g/cm^3^)	-	1.57
Density (g/cm^3^)	-	2.17
Porosity (%)	-	27.65
Solubility in hydrochloric acid (%)	≤2	1.20
Void fraction (%)	≥40	48.68
Specific surface area (m^2^/g)	≥0.5	3.61

**Table 5:** Ion concentrations in high-fluoride wastewater.

Ion	F^−^	Al^3+^	Ca^2+^	Mg^2+^	${\mathrm{SiO}}_{3}^{2-}$	Cl^−^	${\mathrm{SO}}_{4}^{2-}$
Concentration (mg/L)	4000	500	500	500	300	4580	1000

**Table 6:** Fitting parameters of the Langmuir and Freundlich models.

Model	Langmuir			Freundlich		
Parameter	K_L_ (L/mg)	Q_m_ (mg/g)	R^2^	K_F_ (L/mg)	$\frac{1}{n}$	R^2^
298.15 K	0.0057	43.5932	0.9129	4.3203	0.3067	0.9971

**Table 7:** Fitting parameters of kinetic model for F removal by VT-Ceramsite.

Model	Pseudo-First-Order			Pseudo-Second-Order		
Parameter	K_1_ (min^−1^)	Q_e_ (mg/g)	R^2^	K_2_ (g/mg·min^−1^)	Q_e_ (mg/g)	R^2^
298.15 K	0.0018	36.5909	0.9920	0.0196	51.0204	0.9765
308.15 K	0.0019	37.5188	0.9825	0.0193	51.8135	0.9614
318.15 K	0.0022	37.5045	0.9770	0.0203	48.2611	0.9635

**Table 8:** Parameters of Weber-Morris internal diffusion model for fluoride removal by VT-Ceramsite.

Model	Intra-Particle Diffusion					
	Liquid Firm Diffusion		Surface Chemisorption		Particle Diffusion	
Parameter	K_ip_ (g/mg·min^−0.5^)	R^2^	K_ip_ (g/mg·min^−0.5^)	R^2^	K_ip_ (g/mg·min^−0.5^)	R^2^
298.15 K	1.2426	0.9960	0.4868	0.9839	0.0041	-
308.15 K	1.3530	0.9999	0.8708	0.7972	0.0179	0.6947
318.15 K	1.4322	0.9999	1.3026	0.9722	0.0127	0.5549

**Table 9:** Leaching concentration of heavy metals in VT-Ceramsite.

Heavy Metals	Cr	Cd	Pb	Zn	As
Limits (mg/L)	15	1.00	5.00	100	5.00
Concentration (mg/L)	0.67	0.45	<0.01	11.25	1.56

**Table 10:** The adsorption capacity of several adsorbents has been reported in the literature.

Absorbent	Adsorbent Dosage (g/L)	Fluoride Concentration (mg/L)	Q_e_ (mg/g)	Removal Percent (%)	References
Water treatment plant (WTP sludge)	6	5	0.21	28	[39]
Biochar produced from coffee grounds	10	14	0.53	27	[40]
Phosphate tailings	0.4	10	26.89	-	[41]
Weathered coal shale	60	3	23.66	47.19	[42]
Red mud-based geopolymer microspheres (RM@GMs)	1	1.5	76.57	98.88	[43]
Activated sludge lysis ash/chitosan (ASLA/C)	10	10	5.71	89.13	[44]
Ferric modified chromium (III)-fibrous protein (Fe-CrFP)	1	19	14.12	-	[45]
Functional activated carbon	4	4	20	99	[46]
Modified carbon of oak fruit	1	50	26	-	[47]
Rare earth-based adsorbents	2	100	114.47–118.43	-	[48]
VT-Ceramsite	100	4000	43.59/42.15	>90	This work

## Data Availability

The original contributions presented in this study are included in the article. Further inquiries can be directed to the corresponding author.
